# Fast and high temperature hyperthermia coupled with radiotherapy as a possible new treatment for glioblastoma

**DOI:** 10.1186/s40349-016-0078-3

**Published:** 2016-12-08

**Authors:** Giovanni Borasi, Alan Nahum, Margarethus M. Paulides, Gibin Powathil, Giorgio Russo, Laura Fariselli, Debora Lamia, Roberta Cirincione, Giusi Irma Forte, Cristian Borrazzo, Barbara Caccia, Elisabetta di Castro, Silvia Pozzi, Maria Carla Gilardi

**Affiliations:** 1 Department of Medicine, University of Milano Bicocca, Milano, Italy; 2 Physics Department, Liverpool University, Liverpool, UK; 3 Department of Mathematics, College of Science, Swansea University, Swansea, UK; 4 Istituto di Bioimmagini e Fisiologia Molecolare–Consiglio Nazionale delle Ricerche, Palermo, Italy; 5 Istituto Nazionale Neurologico “C.Besta”, Milan, Italy; 6 Istituto Superiore di Sanità, Rome, Italy; 7 Erasmus MC Cancer Institute, Rotterdam, The Netherlands; 8 Sapienza University of Rome, Rome, Italy; 9 Istituto di Bioimmagini e Fisiologia Molecolare–Consiglio Nazionale delle Ricerche, Milan, Italy

**Keywords:** Magnetic resonance-guided focused ultrasound, Hyperthermia, Oncology, Glioblastoma

## Abstract

**Background:**

A new transcranial focused ultrasound device has been developed that can induce hyperthermia in a large tissue volume. The purpose of this work is to investigate theoretically how glioblastoma multiforme (GBM) can be effectively treated by combining the fast hyperthermia generated by this focused ultrasound device with external beam radiotherapy.

**Methods/Design:**

To investigate the effect of tumor growth, we have developed a mathematical description of GBM proliferation and diffusion in the context of reaction–diffusion theory. In addition, we have formulated equations describing the impact of radiotherapy and heat on GBM in the reaction–diffusion equation, including tumor regrowth by stem cells. This formulation has been used to predict the effectiveness of the combination treatment for a realistic focused ultrasound heating scenario.

Our results show that patient survival could be significantly improved by this combined treatment modality.

**Discussion:**

High priority should be given to experiments to validate the therapeutic benefit predicted by our model.

**Electronic supplementary material:**

The online version of this article (doi:10.1186/s40349-016-0078-3) contains supplementary material, which is available to authorized users.

## Background

Glioblastoma (GBM) is a highly aggressive tumor of the central nervous system, corresponding to grade IV of the World Health Organization’s histological classification [1]. High-grade gliomas are the most common primary brain tumors in adults, with an incidence of 3.1 per 100,000 person-years in USA and with a median survival time of 14.6 months after diagnosis [2] and 11.9 months after first resection [3]. Because of their invasive nature, GBMs recur in more than 90% of patients, generally centrally [4] even if marginal and distant failures are reported [5]. The current standard treatment includes external beam radiotherapy (EBRT), maximal surgery, and chemotherapy with temozolomide (TMZ).

The standard treatments for GBM that include EBRT result in a significant increase in patient survival [6]. Dose escalation studies have demonstrated survival improvements up to an overall dose of 60 Gy [7, 8], generally with a dose fractionation of 2 Gy/day, 5 days a week, for a total of 6 weeks for the whole treatment. Beyond this dose, there is only a minimal increase in survival for severely increased toxicity [5, 9].

The study by Elaimy et al. [10] supports the use of stereotactic radiosurgery (SRS). SRS is used either to boost EBRT treatment or to treat small-volume recurrences. The addition of bevacizumab (BEV) after SRS was shown to lower the rate of tumor progression and radio-toxicity [11, 12]. The potential advantages of combining high-intensity focused ultrasound (HIFU) and radiotherapy (RT) in oncology were recently reviewed [13].

In a recent paper, Coluccia et al. [14] described the first successful non-invasive thermal ablation of a brain tumor with transcranial magnetic resonance-guided focused ultrasound (TcMRgFUS) [15]. This paper reported a tumor recurrence in the left thalamic and subthalamic region after surgery for a posteromedial temporal lobe GBM. A total of 25 sonications was applied (17 over the heat ablative threshold); the total sonication time was more than 3 h and about one tenth (0.7 cm^3^) of the total enhancing tumor volume (6.5 cm^3^) was ablated with an Insightec MRgFUS Exablate Neuro system [15].

The main aim of this paper is to demonstrate theoretically, as a “proof of principle”, how the use of TcMRgFUS to generate “fast” hyperthermia (HT), combined with 6 weeks’ EBRT therapy (one or two sessions per week, 1 h each), could have resulted in a successful treatment of the whole tumor. Our approach requires minimal or no modification of the commercially available brain sonication system [15].

## Methods/Design

### Radiotherapy and radiobiology stem cells and the new hypothesis

Several studies have been carried out in order to evaluate the radiation response of human glioma cells. The most recent and complete study on the radiobiological parameters comes from Ferrandon et al. [16]. They analyzed the photon (and carbon ion) response of eleven human-derived glioblastoma cell lines, from the most radio-resistant (T-98G with α = 0.022 Gy^−1^, β = 0.025 Gy^−2^, α/β = 0.9 Gy) to the most sensitive (U-251 with α = 0.630 Gy^−1^, β = 0.019 Gy^−2^, α/β = 35 Gy). Of course, different tumors can have a different cellular composition. Two quite recent papers [17, 18], using different data sets and methodologies, still showed similar data ([17]: α = 0.06 Gy^−1^ ± 0.05 Gy^−1^, α/β = 10 ± 15.1 Gy, while Jones et al. [19] derive the following median values: α = 0.077 Gy^−1^, β = 0.009 Gy^−2^, α/β = 9.32 Gy). Elaborating data from Walker et al. [7], a lower sensitivity (α = 0.027 Gy^−1^, β = 0.0027 Gy^−2^, α/β = 10 Gy) was obtained [20]. Extracted from nine clinical studies, Pedicini et al. [21] obtained quite higher best estimates (α = 0.12 Gy^−1^, β = 0.015 Gy^−2^, α/β = 8 Gy). Note that these latter data include the effect of old and new drugs, such as carmustine (bis-chloroethylnitrosourea (BCNU)) and TMZ.

All these radiobiological data demonstrate the high resistance of glioblastoma to radiation. Still, the data are not sufficient to explain the unsatisfactory clinical results mentioned. In fact, like other tumors, glioblastoma exhibit the capability of an “adaptive response”: the effect of radiation on tumor cells is not only low but becomes increasingly lower as the treatment progresses [22]. There is increasing evidence that solid tumors are hierarchically organized and contain a small population of cancer stem cells (CSCs) [23, 24]. The subpopulation of CSCs has the capability of self-renewal, an unlimited capability of proliferation and a tendency to recur [25], differing from non-stem cells (CDCs). In vitro and in animal experiments showed that the glioma CSCs were significantly more resistant than normal, differentiated cells [26].

### Mathematical modeling of GBM grow and EBRT effect

Around the late 90s [27–30], researchers recognized that the proliferative–infiltrative nature of GBM could be described mathematically by the reaction–diffusion equation [31, 32]. The possibilities offered by MR imaging confirmed the value of this description and demonstrated the significance of the two major parameters in the basic equation, namely proliferation and diffusion [33–35]. The effect of chemotherapy was introduced into the basic equation in 2003 [36] and EBRT in 2007 [20]. Several authors have considered the effects of radiation [37–43]; these approaches all considered only one tissue (i.e., cancer) diffusing into a medium (i.e., healthy brain), without any modification of the environment. Starting with Gatenby and Gawlinsky [44], the tissues (and basic equations) became twofold, representing the tumor and the environment. Their model predicted a previously unrecognized hypocellular interstitial gap at the tumor–host interface that was demonstrated both in vivo and in vitro. To solve this gap, more detailed models, with five or more equations describing the main tumor elements (such as normal, necrotic and hypoxic tumor cells, vascularity, nutrients, etc.) were subsequently proposed [45–51].

### The (Fisher–Kolmogorov) reaction–diffusion equation and tumor growth

A realistic description of GBM evolution involves two phases: first, the cells proliferate to form a small and dense lesion, then they become more diffuse and the reaction–diffusion equation can be applied [27].

The reaction–diffusion equation, including the effect of EBRT, can be written as:

1 $$ \frac{\partial \left(x,t\right)}{\partial t}=\nabla \cdot \left[D\left(\boldsymbol{x}\right)\nabla c\left(\boldsymbol{x},t\right)\right]+\rho \cdot c\left(x,t\right)\cdot \left.\left(1-\frac{c\left(x,t\right)}{c_{\max }}\right)\right)+R\cdot c\left(x,t\right) $$

where *c* (***x***, *t*) denotes the cell density at position ***x*** and time *t*. If B is the domain in which Eq. (1) is solved, the zero flux at the anatomic boundaries implies:

2 $$ \boldsymbol{n}\cdot \nabla \cdot \left[D\left(\boldsymbol{x}\right)\nabla c\left(\boldsymbol{x},t\right)\right]=0\kern0.5em f\kern0.5em  or\kern0.5em \boldsymbol{x}\kern0.5em  on\kern0.5em \partial B $$

where *n* is the unit vector perpendicular to the elementary surface. To solve Eq. (1), an initial condition must be assumed, i.e., *c*(0, ***x***) = *c*_o_ (***x***). *D*(***x***) is the diffusion coefficient [*l* ^2^ *t* ^−1^] and *c*_max_ is the maximum bearable cell concentration in the tumor. A value of *c*_max_ = 4.2 × 10^8^ cells/cm^3^ can be assumed [52], but an order of magnitude of variation in *c*_max_ has been reported [34, 35]. If *D*(***x***) is known in a 3D volume (by using MR and/or positron emission tomography), Eqs. (1) and (2) can be solved numerically at each point of the domain. The cell-killing effect of EBRT is included through the term *R* [*t* ^−1^], which represents the relative change of cell concentration per unit time, at time *t*. For simplicity, we apply the linear-quadratic (LQ) model [53–56]; a derivation of the *R*-term, compatible with this model, is reported in a recent paper [57].

Considering *D* and *R* in Eq. (4) constants, the equation depends on one single scalar radial coordinate (*r*). In the Results section, the curves 1 and 4–6 are obtained by solving this equation in one radial dimension. The tumor proliferation parameter *ρ* is assumed to be 1.2 × 10^−2^, which corresponds to a volume doubling time of 2 months (exponential growth). For the diffusion parameter *D*, a value of 5.83 × 10^−3^ cm^2^/day was previously proposed [20], which is between the values for white matter (*D*_w_ = 1.3 × 10^−3^) and gray matter (*D*_G_ = 5 × *D*_w_) [30]. This equation is solved using the PDEPE function in MATLAB (version R2010a; MathWorks, Natick, MA, USA), which applies an adaptive time-step routine. The relative and absolute tolerances for a stable solution of the equation solver are 10^−6^ and 10^−9^, respectively.

### HIFU and hyperthermia to treat GBM

The present paper builds on the experience gained in the 70s and 90s with the development of the technology of, and the understanding of, the biological aspects obtained with “scanned ultrasound hyperthermia” [58–80] and in particular on “fast/high temperature ultrasound hyperthermia” [67, 73, 76, 79]. This latter technology may have important advantages over standard clinical hyperthermia (1 h at 42.5 °C), such as reduced dependence on perfusion and tumor inhomogeneity and a superior treatment of the tissue near large blood vessels. However, at the time, it failed to find a relevant clinical application. Our tentative explanation is that, in that period, tumor-selective visualization was not available, and the difference in the response of normal versus cancerous tissue was over-emphasized. At higher temperatures, hyperthermia-enhanced perfusion is blocked so that the best discriminating temperature was found to be 42–43 °C [81–83]. However, in the context of heat–radiation synergy, the tumor hyperthermia-enhanced perfusion is a minor effect with respect to other higher temperature cell-killing mechanisms, such as simultaneous or sequential blocking of DNA repair and aerobic and hypoxic direct cell killing [84–88]. The results of four trials [70, 74, 78, 89] of clinical HT are given in Additional file 1.

Two complete reviews on technology for hyperthermia are found in [90] and [91].

TcMRgFUS application is a promising non-invasive modality for neurosurgical intervention, but transmission of ultrasound through the skull constitutes a considerable obstacle, as already shown in the early experiments in 1950s [56]. The previously discussed experience of scanned HT demonstrated the usefulness of a new skull-specific-transduced geometry, helmet-like, with an *f* value (radius/diameter) of about 0.5. Substantial research [92–97], including the advent of MR thermography [98–100] and phased-array transducers [101–103], led to the first pilot clinical trial of GBM ablation [15, 104]. While the temperature distribution in the brain was clearly detectable with MRI, it was impossible to reach ablation because of the limited power provided by the device. Improvements in power output have enabled ablative temperatures to be achieved, but only in a small part of the tumor [14].

In Figure five of Coluccia et al. [14], two sonication pulses with maximum temperatures of 55 °C and 58 °C, respectively, are reported as examples. For both pulses, the length of the “beam on” interval is about 13 s. The first one (*T*_max_ = 55 °C) remained under the ablation threshold (*T*_max_ > 55 °C), while the second one (*T*_max_ = 58 °C) is considered to be an ablative pulse. Both pulses are fitted with Parker’s equation for the pencil beam [105, 106], using a nonlinear least squares method (Leveberg–Marquardt) as explained in the section Additional file 2.

**Fig. 1:**
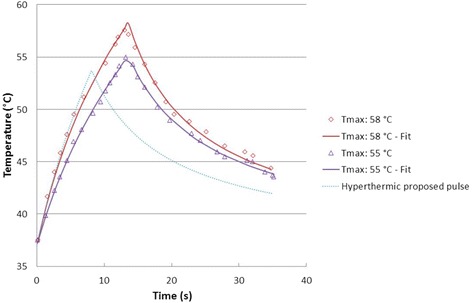
Different HIFU pulses. *Red square*: ablation pulse (*T*_max_ = 58 °C, CEM_43_ > 240 min); *purple triangles*: non-ablation pulse (*T*_max_ = 55 °C, CEM_43_ < 240 min); *dotted curve*: proposed HT pulse (*T*_max_ = 53.7 °C, CEM_43_ = 60 min)

In Fig. 1, we show the two pulse shapes mentioned by Coluccia et al. [14] and a proposed HT pulse shape. In Table 1, fitted values are reported for the following parameters: the instant of maximum temperature (*t*_max_), the maximum temperature (*T*_max_), and the calculated equivalent thermal dose, expressed as cumulative equivalent minutes at 43 °C (CEM_43_). *C* and *D* are the Parker’s model fitted constants.

**Table 1:** Fitted parameters corresponding to the different HIFU pulses: the instant of maximum temperature (*t*_max_), maximum temperature (°C_max_), and the calculated equivalent thermal dose CEM_43_. *C* and *D* are the Parker’s fitted constants (see Additional file 2)

Label	*t*max (s)	*T*max (°C)	CEM_43_	*C*	*D*
58 °C	13.01	57.6	1503.0	3.002	0.168
55 °C	13.14	55.0	199.6	2.287	0.127
HT	8.08	53.7	60.0	3.002	0.127

### Radiation plus hyperthermia response

**Fig. 2:**
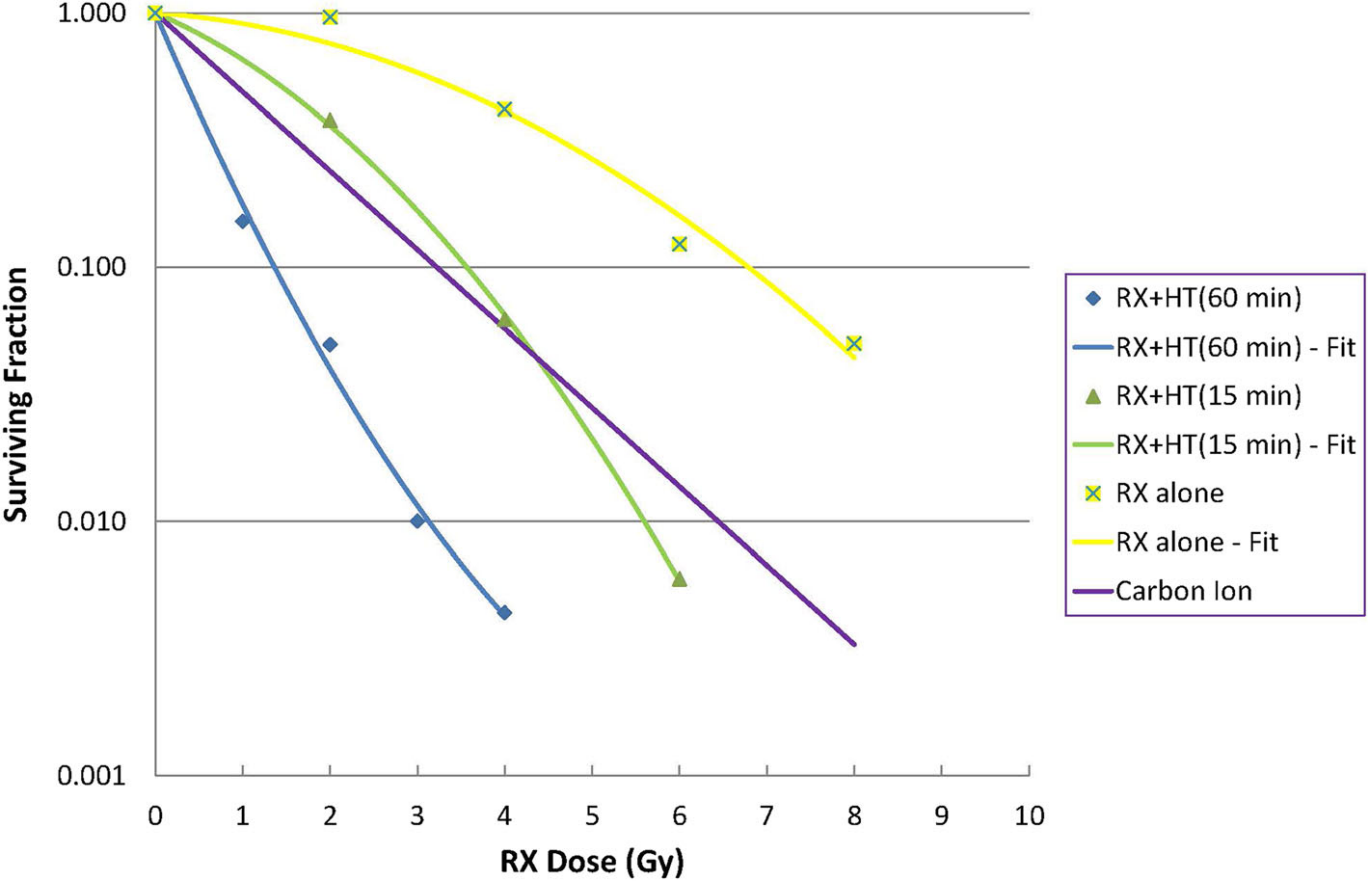
Surviving fraction of U-87MG cells for different radiation qualities. *Yellow crosses*: RX alone (treatment A); *purple line*: carbon ions (curve drawn from Ferrandon et al. [16]); *green triangles*: RX + HT 15 min (treatment B1); *blue diamonds*: RX + HT 60 min (treatment B2).

The three human glioma cell lines (U-87MG, U-138MG, U-373MG) have a large capacity to recover from potentially lethal radiation damage. Since hyperthermia causes radiosensitization and inhibition of recovery from radiation damage, its combination with radiotherapy creates a potent combination for treating human brain tumors [22, 107–109]. In addition, Li et al. [110] and Raaphorst et al. [111] showed that HT has a greater effect on the inhibition of recovery when applied after irradiation with X-rays (RX) compared to before irradiation. In Fig. 2, we report data from Raaphorst et al. [108], in which the surviving fractions (SF) of the U-87MG cells are compared (8-h plating). Two sets of treatment were applied to the cells, radiation only (treatment A) and radiation followed by either 15 min of HT (treatment B1, CEM_43_ = 60 min) or 60 min. HT (Treatment B2, CEM_43_ = 240 min). In both cases, the HT was administered 5 min after the end of the irradiation. For comparison, we have added the carbon ion curve from Ferrandon et al. [16].

As is well known, carbon ions are more effective than RX (the carbon ion RBE at 10% survival is 2.57), but Fig. 2 shows that HT + RT is an extraordinarily effective combination.

Focusing on the RX plus HT 15-min curve (B1 treatment, CEM_43_ = 60 min), we hypothesize, as a first choice, a treatment with 3 Gy per session (SF = 0.167), two sessions per week, 6 weeks of total treatment time, with a total dose of 36 Gy. Each EBRT session should be followed, as soon as possible, by an HT treatment of the whole tumor. From Fig. 5 of Raaphorst et al. [108] and from the chosen protocol, we estimated a decrease of the sensitization effect of about 10%, for a delay of 1 h between the end of EBRT and the beginning of HT. In agreement with Raaphorst et al., we conclude that due to the greater repair capacity of cancer cells, the HT effect is expected to be more effective on tumor than on normal cells. The choice of the proposed protocols is dictated by the desire to maximize the therapeutic ratio of the treatment: reduction of healthy tissue damage but maximization of tumor effect.

Note for the proposed protocols (B1 and B2) the following six points:

1. The total EBRT dose and fractionation should be acceptable, since α and β as previously given will lead to a lower biological effective dose than the “standard protocol” (2 Gy per session, five sessions per week, 6 weeks total treatment time. Maximum total dose is 60 Gy)
2. Exploiting the time interval of 2–3 days between the treatments, the phenomenon of thermo-resistance due to heat shock proteins (HSP) that can reduce the treatment effect, is avoided [112]
3. The induction of the immunogenic tumor cells and direct tumor cell killing by HT in combination with EBRT can contribute to immune activation against the tumor [113]
4. Thanks to the nearly constant sensitivity to RX plus HT of CSCs and CDCs found for the cell lines evaluated [114], the problem of resistant sub-populations should be avoided
5. The effect of (mild) HT on hypoxic tumor regions is well known [115, 116], and there are several indications that this effect is similar or stronger at higher temperatures [117, 118]
6. In treatment B2, only one session per week is required, given the increased dose (4.4 Gy × 6)

Clearly, the main limitation of the treatment described in Coluccia et al. [14] is the time required to alleviate the pain to the brain of the patient, requiring long cooling intervals between the sonication. This pain is mainly due to the heat energy absorbed by the bone (30–60 times more than by the soft tissue [119]). With the proposed HT pulses, the warming of the whole T1w-enhancing tumor would require 163 pulses, for a total time of 1.27 h (each pulse consisting of 8.1 s of beam on and 20 s of “beam off”, i.e., cooling down time). The cooling down interval has been here calculated on the basis of HIFU ablative literature [14, 120, 121] and still requires experimental verification. Let us consider another example: a tumor with an equivalent radius of 2.3 cm (volume of 51 cm^3^) of which about 92% of the volume was removed by surgery. The residual tumor, i.e., our target, is therefore 4.2 cm^3^. This requires about 105 sonications with 8.1 s of beam on time. The total treatment time would be less than 1 h (49.2 min).

## Results

**Fig. 3:**
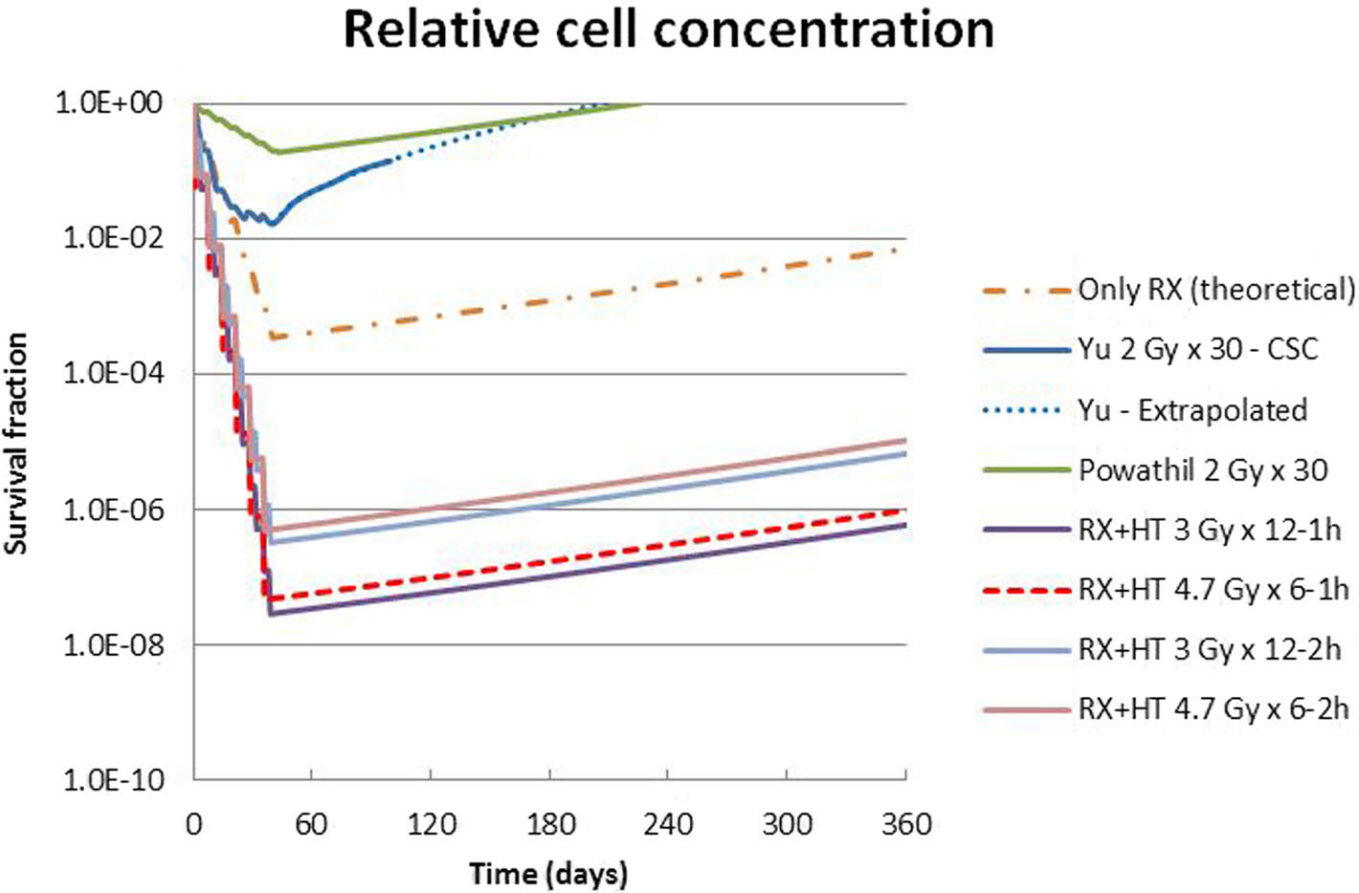
Comparison of relative glioblastoma cell survival by using different treatments, as illustrated in Table 2

In this section, we compare the tumor cell survival corresponding to the different treatments (Table 2 and Fig. 3).

**Table 2:** Parameter values along with bibliographic references of the curves shown in Fig. 3. “Time to offset” is the time for the tumor to come back to the initial value. Curves n. 5 and 6 are calculated with 1-h delay between the end of RT and the beginning of HT. Curves 7 and 8 are calculated with 2-h delay between the end of RT and the beginning of HT

Number	Title	α (Gy^−1^)	β (Gy^−2^)	Minimum survival value	Time to offset	Reference
1	Only RX (unrealistic)	5.4 × 10^−2^	4.2 × 10^−2^	3.61 × 10^−4^	2.29 years	[108]
2	Yu 2 Gy × 30–CSCs	1 × 10^−2^ 1.25 × 10^−1^	1.77 × 10^−7^ 2.8 × 10^−2^	1.66 × 10^−2^	210 days	[122]
3	Yu–Extrapolated	1 × 10^−2^ 1.25 × 10^−1^	1.77 × 10^−7^ 2.8 × 10^−2^	–	–	Extrapolation of the previous SF
4	Powathil 2 Gy × 30	2.7 × 10^−2^	2.7 × 10^−3^	1.89 × 10^−1^	226 days	[20]
5	RX + HT 3 Gy × 12–1 h	3.36 × 10^−1^	8.7 × 10^−2^	2.897 × 10^−8^	4.61 years	[108]
6	RX + HT 4.7 Gy × 6–1 h	3.36 × 10^−1^	8.7 × 10^−2^	4.986 × 10^−8^	4.48 years	[108]
7	RX + HT 3 Gy × 12–2 h	3.36 × 10^−1^	8.7 × 10^−2^	3.286 × 10^−7^	4.02 years	[108]
8	RX + HT 4.7 Gy × 6–2 h	3.36 × 10^−1^	8.7 × 10^−2^	4.986 × 10^−7^	3.91 years	[108]

Note that the “standard” treatment (2 Gy × 30, N.1 in Table 1, dash-dotted orange line in Fig. 3) is calculated neglecting the stem cell effect (only EBRT–RX). Due to the presence of cancer stem cells, this treatment should be considered unrealistic.

Curves 2 and 3 (solid and dotted blue lines, respectively, in Fig. 3) include the effect of the CSC cells [122]. The exceptional resistance of CSCs and the interplay of these cells with CDC progressively reduces the rate of decrease of the cancer cells (“adaptive response”). The clinical data are reproduced reasonably well by the LQ model when assuming lower α and β values (curve 4, solid green line, in Fig. 3). This model is applied to both CSCs and CDCs. The fraction of CSCs in the total cell number (*F*) is assumed to be 1.6 × 10^−2^. Curve 4 was previously calculated [20] using Eq. (4) and clinical radiobiological data [7]. It is interesting to note that this curve, by assuming very low values for α and β, reproduces quite well the “time to offset” (i.e., the time for the tumor volume to grow back to its initial volume) of the previous curves that are based on the effect of CSCs. Curves 5 and 6 in Table 2 (purple solid and red dotted lines in Fig. 3) show the effect of the new RX + HT treatments. Curve 5 corresponds to protocol B1, with two sessions a week (for example, Monday and Thursday), in which, after conformal RT with a maximum dose of 3 Gy, HT is administered with a CEM_43_ of 60 min. Curve 6 corresponds to protocol B2: one session a week with a larger radiation dose (4.7 Gy) and the same thermal dose (CEM_43_ = 60 min). Curves n. 5 and 6 are calculated with 1-h delay between the end of RT and the beginning of HT. Curves 7 and 8 are calculated respectively as curves n. 5 and 6, but with 2-h delay between the end of RT and the beginning of HT.

## Discussion and conclusions

As is clear from Table 2 and Fig. 3, the proposed protocols with RX + HT easily outperform the traditional ones; they lead to a low survival level of tumor cells and a long offset time such that the patient is effectively cured, in contrast to glioblastoma treatment today where cure is never achieved. At very low levels of cell survival, the immune system may also play an important role [123]. To our knowledge, the proposed methodology is the only one capable of achieving curative outcomes. In addition, we wish to emphasize that we used the CEM_43_ concept [124] to establish an equivalence, in terms of the total cell-killing effect, between the Raaphorst data [108] obtained at 45 °C and the hypothesized HT pulse shape of Fig. 1. This choice doesn’t take into account radiation–heat synergy. In this regard, Law [125] found that for heat combined with X-rays, the time required to produce a given level of radiodermatitis was reduced by a factor of three for a rise in temperature of 1° (in comparison with the Sapareto and Dewey [124] law, which predicts a factor of two). Even if the law result was obtained in a different tissue, our data could be an underestimation, and a pulse shape with a lower HT maximum temperature and/or a lower time length can be possible in practice. Of course, on this point, direct experimental data are required. In any case, the illustrated results can be obtained without significant modification of the present system. Regarding the two proposed protocols, only experience will help to choose between them: B2 is easier to execute in clinical practice (just one session a week) but it is not clear if we would have the same benefits from the clinical point of view (oxygenation and immune system stimulation).

It is important to emphasize that the equivalent doses [56] of the two proposed treatments are more than 35% lower than the “reference” dose of 60 Gy, given in 2 Gy fractions, five days a week for a total time of 6 weeks. This very important consequence of the proposed scheduling would reduce drastically the radiation damage to surrounding healthy tissues, evaluated in terms of normal tissue complication probabilities [126]. To achieve a uniform treatment of the target volume, as for ablation, the HT spots can be spaced in a raster by superimposing the isolines (or, better, isosurfaces) 50% of the thermal dose. There is no reason for the 50% isolines of our HT to have a different shape or location compared to the ablation treatment (Additional file 3).

The main limitation of our proposed treatment is that the tumor volume assumed in our modeling only measures about 4–6 cm^3^. However, in the light of the groundbreaking results described in this paper, maximum effort must be made to extend the size of the tumor mass that we can cure. It can be noted that we haven’t considered methods for drastically lowering the frequency (220 kHz) to open the blood–brain barrier (BBB) and improve drug delivery [127–130], with the use of microbubbles [131, 132], nanoparticles [133–135], or contrast media [136]. Such a strategy is highly problematic [137, 138].

The proposed treatment can increase the treated region by using at least two different techniques.

Firstly: in the presence of radiation, the Sapareto–Dewey law [124] may no longer be valid, and a different model should be used to establish the time–temperature equivalence. If we follow the Law [125] expression (for every 1° increase, one third (not a half) of the time required for the same effect), the maximum temperature of the HT pulse in Fig. 1 would be less than 50 °C, and the useful impulse length would be about 6 s instead of about 8 s (for the same effect). In addition, a lower temperature HT pulse would require a lower ultrasonic (US) intensity, which means a lower pressure. In turn, a lower pressure on the skull would allow, at the same level of safety with respect to a possible cavitation event, a lower frequency. This latter would give a better US transmission and a lower temperature on the skull, thereby reducing the cooling time and allowing a larger target volume to be treated.

Secondly: making better use of the heat that flows, inside the target, from higher to lower temperature regions. In this regard, different methods are described in the literature of covering the tumor volume [139–143] more efficiently than the point-by-point strategy adopted here for simplicity.

In addition, it is important to consider correctly the evolution of GBM. All the three more complete GBM models mentioned in the text [47, 48, 51] predict that in the evolution of the disease, the biologically active region (proliferating and infiltrating) is pushed toward the periphery of the tumor, while the central part becomes progressively larger and necrotic. Therefore, it seems reasonable to concentrate in this peripheral region both radiation and HT. This would change radically the GBM treatment planning and would reduce significantly the region to be heated (and irradiated) [144].

In light of the above, there is significant room for improvement of the proposed technique.

As has been emphasized in relation to the results obtained, these new implementations also require careful experimental validation, but the door is open for a truly effective and, possibly, life-saving GBM treatment.

Addendum: this paper is dedicated, in particular, to our friends Mario Granata and Luciano Andreucci, who died from this devastating disease.

## Additional files

Additional file 1: Reports the results of four trials of clinical HT. (DOCX 18 kb)
Additional file 2: Reports US pulses fitted with the Parker's equation, using nonlinear least squared method. (DOCX 21 kb)
Additional file 3: Location of isolines in ablative and HT US pulses. (DOCX 19 kb)

## Abbreviations

BCNU: Bis-chloroethylnitrosourea
BEV: Bevacizumab
CDCs: Non-cancer stem cells
CEM_43_: Cumulative equivalent minutes at 43 °C
CSCs: Cancer stem cells
EBRT: External beam radiotherapy
FUS: Focused ultrasound
GBM: Glioblastoma
HIFU: High-intensity focused ultrasound
HT: Hyperthermia
RT: Radiotherapy
RX: X-rays
SRS: Stereotactic radiosurgery
TcMRgFUS: Transcranial magnetic resonance-guided focused ultrasound
TMZ: Temolozomide
