# Supplementary material for: Fast and high temperature hyperthermia coupled with radiotherapy as a possible new treatment for glioblastoma
Source: J Ther Ultrasound. 2016 Dec 8;4:32. doi: 10.1186/s40349-016-0078-3 (PMC5143464; doi:10.1186/s40349-016-0078-3)
Supplement: Additional file 1: — Reports the results of four trials of clinical HT. (DOCX 18 kb) [file 40349_2016_78_MOESM1_ESM.docx]

# Additional file 1: Reports the results of four trials of clinical HT

The clinical results of a phase I trial of the treatment of malignant brain tumours with scanning HT, generated by the device described in [65] with methods reported in [89] is discussed in the Guthkelch et al. paper [70]. The hyperthermia was administered weekly with EBRT given 5 times a week to 15 recurrent patients with craniotomy and multiple thermal sensors inserted into the brain. The target temperature was 42.5 °C at the tumour boundary, while the internal maximum temperature was not allowed to exceed 46 °C. The scanning was modeled on concentric octagons in each plane to cover a tumor volume up 5 x 4 x 4 cm at a depth not exceeding 6 cm below the Dura level. The protocol contemplated a time escalation of 30, 45 and 60 mins. Technical problems and toxicities hampered the trial due, in particular to the initial patient status, clinical problems related to the insertion of a catheter with sensors, surgical use of unsuitable Dural substitutes, tumour location in the temporal lobe and high perfusion lesions. However all 5 autopsied patients showed clear evidence of treatment-induced necrosis of their tumours.

A randomized phase II/III trial [74] (Stea et al, 1992) of newly diagnosed patient were treated with conventional EBRT (59.4 Gy with daily fraction of 1.8 Gy) then, 2 weeks after EBRT, 33 of them had a brachytherapy boost with the insertion of a certain number (2 – 6) of silastic catheters, loaded with a radioactive source of 125 Iodine (I-125) with an activity in the range of (10 – 20 mCi); these were removed after a delivery of 60 Gy at the reference isodose. The other group of patient (35) received, in addition, interstitial HT by using helical coil antennas working at 915 MHz. HT was given 30 min before and 30 min after the radiation boost (the same catheters were used for radioactive sources, then replaced with HT antennas and a second time with I-125. Antennas were spaced 1.2 – 1.8 cm from each other and one or two additional catheters were loaded with sensors (or left empty in the first group). The protocol stipulated that the tumour be heated to at least 42.5 °C, without exceeding 50 °C. The thermal dose was evaluated by calculating T90 and T50 (that means the CEM to the 90 and 50% of the tumour volume). The median survival of the HT group was 85 weeks vs 76 weeks and the 2 year survival was 31% versus 15 %. The toxicity was judged acceptable.

Another Phase I/II clinical trial [78] (Sneed, 1998) compared the survival of two groups of patients with supratentorial malignant gliomas who were treated on two sequential protocols with either interstitial thermoradiotherapy (25 patients) or with interstitial irradiation without hyperthermia (37 patients). The treatment consisted of debulking surgery, a course of EBRT plus HT given immediately before and after brachytherapy. The survival of this group was compared with a similar group treated with interstitial brachytherapy alone. The interstitial hyperthermia was given by means of ferromagnetic seeds strung together in ribbons which were afterloaded into the surgically placed catheters. The ferromagnetic seeds or implants were heated by an externally applied radiofrequency magnetic field, induced by a magnetic induction coil. The hyperthermia treatment was given for 60 min before and immediately after removal of the radioactive sources. The aim was to heat the target volume to temperatures between 42°C and 45°C. Temperatures were continuously monitored with multisensor thermocouple probes, or fiberoptic thermometers. The interstitial thermoradiotherapy boost gave a statistically significant survival benefit when compared to interstitial brachytherapy alone. The survival curves of the two groups were well separated and, in particular, at 24 months the survival of the first group was 48 %, compared to 18% for the group of brachytherapy only. The morbidity was acceptable.

More recently [89] (Maier-Hauff, 2011), intratumoral thermotherapy using magnetic iron-oxide nanoparticles, combined with reduced dose EBRT on 59 recurrent GBM patients was tried. The patients underwent a neuro-navigation-controlled injection of iron oxide nanoparticles and a subsequent heating with alternating magnetic fields. The treatment was combined with fractionated EBRT (30 Gy). The aim was to achieve a temperature no higher than 43 °C beyond a 2 cm margin around the tumour. After recurrence, the median survival was 13.4 months. The technique was proven to be safe and resulted in a statistically longer survival.
