# Supplementary material for: Fast and high temperature hyperthermia coupled with radiotherapy as a possible new treatment for glioblastoma
Source: J Ther Ultrasound. 2016 Dec 8;4:32. doi: 10.1186/s40349-016-0078-3 (PMC5143464; doi:10.1186/s40349-016-0078-3)
Supplement: Additional file 2: — Reports US pulses fitted with the Parker's equation, using nonlinear least squared method. (DOCX 21 kb) [file 40349_2016_78_MOESM2_ESM.docx]

**Additional file 2: Reports US pulses fitted with the Parker’s equation, using nonlinear least square method.**

In Fig. 5 of Coluccia et al. [[1](#_ENREF_1)] are reported, as examples, two sonication pulses with, respectively, maximum temperature of 55°C and 58°C (for both pulses, the length of the “beam on” time was about 13s). The first one (T_max_ = 55°C) is demonstrated to be under the ablation threshold (T_max_ > 55°C), while the second one (T_max_ = 58 °C) is considered an ablative pulse. Both pulses are fitted here with Parker’s equation for the pencil beam [[2](#_ENREF_2),[3](#_ENREF_3)]:

$T(t)=\left\{ \begin{aligned} \frac{C}{D}\cdot ln(1+D\cdot t), &t\leq t_{0} \\ \frac{C}{D}\cdot ln\left[ \frac{\left( 1+D\cdot t \right)}{1+D\cdot{(t-t}_{0})} \right], &t>t_{0} \end{aligned} \right.$ (1)

where T(t) is the temperature variation in the beam focus. The upper equation describes the temperature increase during the phase of “beam on” (t ≤ t_0_), while the lower one represents the subsequent decrease during the “beam off” phase (t > t_0_). The constant C [°C/s] is related to the medium absorption coefficient α [m^-1^], the beam intensity I_0_ [J/s^.^m^2^], the medium density δ [kg/m^3^] and the specific heat coefficient at constant pressure c_p_ [J/kg·°C], by the following expression:

$C=\frac{2\cdot\alpha\cdot I_{0}}{\delta\cdot c_{p}}$. (2)

D [1/s] depends on the thermal diffusivity κ [m^2^/s] and β [m^2^], the (Gaussian) squared beam radius (r_0_) divided by 2 (β = r_0_^2^/2), and is given by the equation:

$D=\frac{4\cdot\kappa}{\beta}$ . (3)

The dimension of the C/D ratio is, correctly, a temperature [°C]. In our fit, C and D are free parameters.

[1] Coluccia D, et al. First noninvasive thermal ablation of a brain tumor with mr-guided focused ultrasound. *Journal of therapeutic ultrasound* 2014;2:17.

[2] Dillon CR, et al. An analytical solution for improved hifu sar estimation. *Phys Med Biol* 2012;57:4527-4544.

[3] Parker KJ. Effects of heat conduction and sample size on ultrasonic absorption measurements. *The Journal of the Acoustical Society of America* 1985;77:719-725.
