# Supplementary material for: Fast and high temperature hyperthermia coupled with radiotherapy as a possible new treatment for glioblastoma
Source: J Ther Ultrasound. 2016 Dec 8;4:32. doi: 10.1186/s40349-016-0078-3 (PMC5143464; doi:10.1186/s40349-016-0078-3)
Supplement: Additional file 3: — Location of isolines in ablative and HT US pulses. (DOCX 19 kb) [file 40349_2016_78_MOESM3_ESM.docx]

## Additional file 3: location of isolines in ablative and HT US pulses.

In the transverse plane temperature change profile during focused ultrasound heating, *T* (°C), as a function of radial position *r* (mm) from the beam axis and time *t* (s) since the onset of heating, assuming a Gaussian power distribution with no axial conduction, uniform thermal properties, and perfusion modeled as a scalar thermal energy sink [[1](#_ENREF_1)], is expressed by [[2](#_ENREF_2)]:

$T_{a}(r,t)={IHR}_{a}\cdot\int_{0}^{t} e^{-\frac{t^{'}}{\boldsymbol{\tau}_{\boldsymbol{bl}}}}\cdot\frac{e^{\left( \frac{-\frac{r^{2}}{\beta}}{1+\frac{t^{'}}{\tau_{c}}} \right)}}{\left( 1+\tau_{c} \right)}\cdot dt'$ , (1)

where IHR is the initial heating rate (°C s^−1^) and *β* is the Gaussian variance (mm^2^) of the ultrasound beam. The perfusion time constant *τ*_bl_ (s) is related to the Pennes’ perfusion parameter *w* (kg m^−3^ s^−1^), tissue density *ρ* (kg m^−3^), and specific heats of tissue *c*_p_ and blood *c*_bl_ (J kg^−1^ °C^−1^) by the equation *τ*_bl_ = *ρc*_p_/(*wc*_bl_). The conduction time constant *τ*_c_ (s) depends upon tissue thermal diffusivity *α* (mm^2^ s^−1^) and *β* according to *τ*_c_ = *β*/(4*α*).
To our knowledge, the full analytical solution of (1) cannot be directly evaluated. For HT, is valid exactly the same equation (1), so that:

$\frac{T_{a}(r,t)}{T_{h}(r,t)}=\frac{{IHR}_{a}}{{IHR}_{h}}=\frac{C_{a}}{C_{h}}$ (2)

Where the indexes a and h indicate, respectively, ablation and hyperthermia: the ratio between the initial heating rates is the same as the ratio between the fitted parameters C, relatives to ablation and hyperthermia, reported in Table 1 of the text.

As reported in Table 1, the two values of C relatives to ablative and hyperthermic pulses have the same values, so that the corresponding temperature levels, respect to maximum (r = 0) are the same. Considering the 2π geometry of the helmet, a similar reasoning may, reasonably, be applied also to the axial direction. In other words, the ellipsoidal shape of the focal region (respect to the maximum) is the same both for ablative and hyperthermic pulses.

1. Pennes, H.H., *Analysis of tissue and arterial blood temperature in the resting forearm.* Journal of Applied Physiology, ed. A.P. Society. Vol. 1. 1948: American Physiological Society.

2. Cline, H.E., et al., *MR temperature mapping of focused ultrasound surgery.* Magn Reson Med, 1994. **31**(6): p. 628-36.
